# Anabolic deficits and divergent unfolded protein response underlie skeletal and cardiac muscle growth impairments in the Yoshida hepatoma tumor model of cancer cachexia

**DOI:** 10.14814/phy2.70044

**Published:** 2024-09-18

**Authors:** Daniel J. Belcher, Nina Kim, Blanca Navarro‐Llinas, Maria Möller, Francisco J. López‐Soriano, Silvia Busquets, Gustavo A. Nader

**Affiliations:** ^1^ Huck Institutes of the Life Sciences The Pennsylvania State University University Park Pennsylvania USA; ^2^ Department of Kinesiology The Pennsylvania State University University Park Pennsylvania USA; ^3^ Department of Biochemistry and Molecular Medicine University of Barcelona Barcelona Spain; ^4^ Institut de Biomedicina de la Universitat de Barcelona Barcelona Spain; ^5^ Penn State Cancer Institute The Pennsylvania State University University Park Pennsylvania USA

**Keywords:** cancer, inflammation, ribosomal RNA, unfolded protein response, wasting

## Abstract

Cancer cachexia manifests as whole body wasting, however, the precise mechanisms governing the alterations in skeletal muscle and cardiac anabolism have yet to be fully elucidated. In this study, we explored changes in anabolic processes in both skeletal and cardiac muscles in the Yoshida AH‐130 ascites hepatoma model of cancer cachexia. AH‐130 tumor‐bearing rats experienced significant losses in body weight, skeletal muscle, and heart mass. Skeletal and cardiac muscle loss was associated with decreased ribosomal (r)RNA, and hypophosphorylation of the eukaryotic factor 4E binding protein 1. Endoplasmic reticulum stress was evident by higher activating transcription factor mRNA in skeletal muscle and growth arrest and DNA damage‐inducible protein (GADD)34 mRNA in both skeletal and cardiac muscles. Tumors provoked an increase in tissue expression of interferon‐γ in the heart, while an increase in interleukin‐1β mRNA was apparent in both skeletal and cardiac muscles. We conclude that compromised skeletal muscle and heart mass in the Yoshida AH‐130 ascites hepatoma model involves a marked reduction translational capacity and efficiency. Furthermore, our observations suggest that endoplasmic reticulum stress and tissue production of pro‐inflammatory factors may play a role in the development of skeletal and cardiac muscle wasting.

## 1 INTRODUCTION

Cachexia is a multifaceted wasting condition that increases overall mortality and morbidity of cancer patients (Argilés et al., 2014; Baracos et al., 2018; Orellana López et al., 2023). Reductions in fat and skeletal muscle mass and strength are hallmarks of this condition (Argilés et al., 2014; Baracos et al., 2018). Also prevalent is the loss of cardiac mass, which leads to impaired cardiovascular function and reduced physical capacity (Barkhudaryan et al., 2017). Collectively, these deteriorations severely reduce quality of life and shorten lifespan of cancer patients (Argilés et al., 2014; Baracos et al., 2018; Barkhudaryan et al., 2017). Hepatocellular carcinoma is one of the fastest growing and deadliest forms of cancer in the U.S. (McGlynn et al., 2021) with approximately 25% of patients developing cachexia (Wolf et al., 2021). The Yoshida AH‐130 ascites hepatoma is one of the most extensively studied preclinical models of cachexia. Rats carrying AH‐130 tumors experience cachexia (Baracos et al., 1995; Salazar‐Degracia et al., 2018; Toledo et al., 2014; Yuan et al., 2023) with elevated systemic and local inflammation (Cernackova et al., 2019; Costelli et al., 1995), disrupted cellular energy metabolism (Barreiro et al., 2005; Toledo et al., 2016), and elevated proteolysis (Barreiro et al., 2005; Toledo et al., 2014, 2016). Cachexia may also results from a disruption in the intrinsic balance between protein synthesis and degradation, that is protein turnover (Tessitore et al., 1993), whether from a reduction in protein synthesis, an elevation in protein degradation, or a combination of both. While the mechanism involved in muscle proteolysis are well defined, less is known about anabolic deficits in cachexia. We recently showed that reduced anabolism underlies muscle wasting in ovarian, lung and colon cancer models (Belcher et al., 2022; Kim et al., 2021, 2022). However, whether this mechanism is consistent in the heart and in other models, cancer cachexia models remains to be determined. In addition to the reduction in ribosomal capacity (i.e. translational capacity), defective translational efficiency may also play a significant role in the maintenance of muscle mass because alterations in translational control can result from endoplasmic reticulum (ER) stress, a response supporting the restoration of cellular proteostasis (Preston & Hendershot, 2013). ER stress‐induced unfolded response activation is commonly observed in several conditions such as sarcopenia, inflammatory myopathies, muscular dystrophy, and cancer cachexia (Bohnert et al., 2018). Whether ER stress is involved in skeletal and cardiac muscle wasting in the Yoshida AH‐130 hepatoma model is unknown.

The goal of this study was to determine whether reductions in translational capacity and efficiency underlie skeletal and cardiac muscle wasting in the Yoshida AH‐130 hepatoma model, and whether the induction of the unfolded protein response (UPR) and local expression of pro‐inflammatory cytokines were modulated in this model of cachexia. We demonstrate that inoculation with Yoshida AH‐130 tumor cells caused a reduction in ribosomal capacity and 4E binding protein 1 (4E‐BP1) phosphorylation in both skeletal and cardiac muscle. Wasting was associated with activating transcription factor (ATF4) and GADD34 gene expression in skeletal muscle, but the latter only in the heart. Local interleukin (IL)‐1β expression was detected in both skeletal and cardiac muscle of cachectic rats and was accompanied by a marked increase in IFN‐γ expression in the heart but not in skeletal muscle. Overall, these findings suggest that Yoshida AH‐130 tumor‐bearing rats undergo skeletal and cardiac muscle wasting, in part, via a reduction in translational capacity, dysregulated translational control signaling, UPR induction, and the expression of tissue pro‐inflammatory cytokines.

## 2 METHODS

### 2.1 Animals and tumor inoculation

Male Wistar rats (8‐week‐old) were housed in a controlled environment with a temperature of 22 ± 2°C and a normal dark cycle of 12 h with lights on from 08:00 am to 08:00 pm. Throughout the duration of the study, they had access to both food (B.K. Universal G.J./S.L., Sant Vicenç del Horts, Barcelona, Spain) and water ad libitum. All animal experiments were made in accordance with the European Community guidelines for the use of laboratory animals (European Parliament Council of the European Union, 2010), and The Bioethical Committee of the University of Barcelona approved the experimental protocol. Rats were randomly divided into control (*n* = 6) or tumor groups (*n* = 7). Animals in the tumor group received an intraperitoneal inoculation of 10^8^ AH‐130 Yoshida ascites hepatoma cells, while those in the control group received an equal volume of saline. After 7 days, animals were weighed and anesthetized with an i.p. injection of ketamine/xylazine mixture (3:1) (Imalgene® and Rompun®, respectively). While under deep plane anesthesia, tumors were carefully extracted from the peritoneal cavity, gastrocnemius muscles and heart tissues were rapidly excised, weighed, frozen in liquid nitrogen, and stored at −80°C until further analysis.

### 2.2 RNA extraction and quantitative polymerase chain reaction

Total RNA was extracted from gastrocnemius and heart tissues as previously described (Belcher et al., 2022). Briefly, tissues were homogenized in TRIzol reagent (Cat. No. 15596026, Ambion, Thermo Fisher Scientific, Waltham, MA), RNA was extracted using Direct‐zol RNA MiniPrep kit (Cat. No. R2051, Zymo Research, Irvin, CA) following the manufacturer's instructions. Total/ribosomal RNA was quantified spectrophotometrically using a CLARIOstar Microplate Reader and a LVis Plate (BMG Labtech, Ortenberg, Germany). Next, cDNA was synthesized from 1ug of RNA through reverse transcription PCR using the SuperScript™ IV VILO™ cDNA synthesis kit (Cat. No. 11756500, Invitrogen, Thermo Fisher Scientific, Waltham, MA). Analysis of mRNA was performed via qPCR (BioRad CX384) with GoTaq qPCR Master (Cat. No. A6002, Promega, Madison, WI) and primers listed in Table 1. All transcripts were normalized to the average of GAPDH and β‐actin by the comparative Ct (ΔΔCt) method and represented as fold‐change from controls. There was no difference between groups in the geometric mean expression of the housekeeping genes. The spliced form of the X‐box‐binding protein 1 (s‐XBP1) was determined as described previously (Bin et al., 2019) using primers targeting the 26 base pair intron cleaved by the Inositol‐requiring enzyme type 1 (IRE1) and normalized to total XBP1 (t‐XBP1) mRNA.

**TABLE 1:** Primers used in this study.

Target	Forward	Reverse
ATF4	GTTTGACTTCGATGCTCTGTTTC	GGGCTCCTTATTAGTCTCTTGG
GADD34	CTCTGAAGGGTAGAAAGGTGC	TCGATCTCGTGCAAACTGCT
s‐XBP1	GCTGAGTCCGCAGCAGGT	CAGGGTCCAACTTGTCCAGAAT
t‐XBP1	TGGCCGGGTCTGCTGAGTCCG	ATCCATGGGAAGATGTTCTGG
IL‐1β	CAGCTTTCGACAGTGAGGAGA	TGTCGAGATGCTGCTGTGAG
IL‐6	CTCTCCGCAAGAGACTTCCA	GGTCTGTTGTGGGTGGTATCC
IFN‐γ	GCCCTCTCTGGCTGTTACTG	CCAAGAGGAGGCTCTTTCCT
TNF‐α	GATCGGTCCCAACAAGGAGG	CTTGGTGGTTTGCTACGACG
GAPDH	AGTGCCAGCCTCGTCTCATA	CGTTGAACTTGCCGTGGGTA
β‐Actin	CACCCGCGAGTACAACCTTCT	CGTCATCCATGGCGAACTGGT

### 2.3 Western blot analysis

All tissue samples were homogenized and extracted in RIPA lysis buffer (50 mM Tris pH 8.0, 150 mM NaCl, 1% Triton X‐100, 0.1% SDS) supplemented with Pierce Protease and Phosphatase Inhibitor Mini Tablets (Cat. No. A32959, Thermo Fisher Scientific, Waltham, MA) using 1 tablet per 10 mL of lysis buffer. Protein (10–30 μg) was separated by SDS‐page on polyacrylamide gels, transferred to PVDF membranes as previously described (Belcher et al., 2022), and immunoblotted using the following antibodies: 4E‐BP1 [1:4000, Cell Signaling Technology (CST); Cat. No. 9644, RRID:AB_2097841]; Phospho‐S6 Ribosomal Protein (S235/236; 1:1000, CST; Cat. No. 2211, RRID:AB_331679), S6 Ribosomal Protein (1:2000, CST; Cat. No. 2217, RRID:AB_331355), eIF4E (1:2000, CST; Cat. No. 2067 RRID:AB_2097675), and Peroxidase AffiniPure™ Goat Anti‐Rabbit IgG (1:10,000, Jackson ImmunoResearch; Cat. No. 111‐035‐144, RRID:AB_2313567). All blots were developed using the Clarity Western ECL Substrate kit (Cat. No. 1705061, BioRad) and verified for equal loading using Ponceau S (Cat. No. J63139.K2, Thermo Fisher Scientific, Waltham, MA).

### 2.4 Statistical analysis

All data are reported as means ± standard deviation (SD) or mean standard error (SEM) where appropriate. Potential outliers were identified by a ROUT test with the maximum false discovery rate set to 1%. All comparisons were analyzed via a two‐tail unpaired *t*‐test. *p*‐values ≤0.05 were considered significant. Correlations between the changes in skeletal or cardiac muscle mass and rRNA were calculated using Pearson's correlation coefficients. All statistical analyses were performed and graphically represented using GraphPad Prism 10 (GraphPad Prism, San Diego, CA, RRID:SCR_002798).

## 3 RESULTS

### 3.1 Yoshida AH‐130 ascites‐induced skeletal and cardiac muscle wasting involves a reduction in ribosomal capacity

**FIGURE 1:**
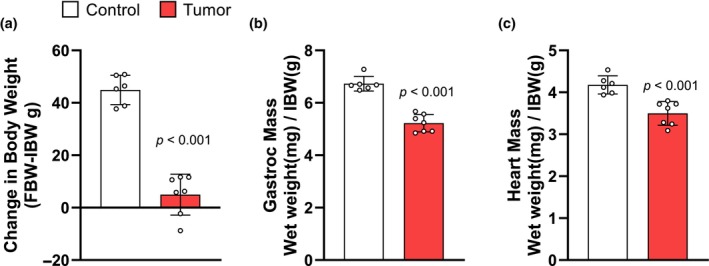
Yoshida AH‐130 tumor inoculation yields a loss in body weight, skeletal muscle, and heart mass. (a) Change in tumor‐free body weight (FBW) relative to initial body weight (IBW) in grams for control and tumor‐bearing rats. (b) End‐point gastrocnemius muscle mass in milligrams was normalized to IBW in grams. (c) Heart mass in milligrams was normalized to IBW in grams. Data are expressed as the mean ± SD. Statistical significance was determined by a two‐tailed unpaired *t*‐test, *p* = significant differences from control. gastroc, gastrocnemius.

**FIGURE 2:**
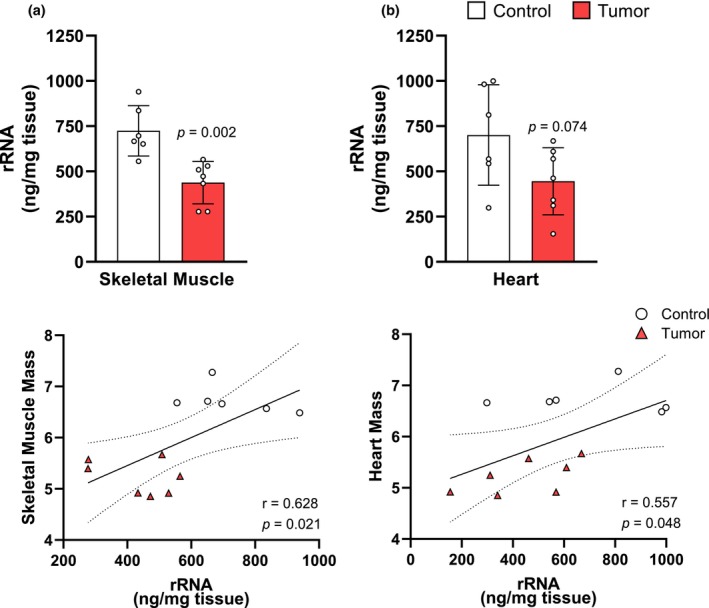
AH‐130 tumor inoculation induces a loss in rRNA that is associated with reductions in skeletal and cardiac muscle mass. (a) Quantification of total RNA in skeletal muscle (top). Pearson's correlation coefficient between rRNA and skeletal muscle mass (bottom). (b) Quantification of total rRNA in the heart (top). Pearson's correlation coefficient between rRNA and heart mass (bottom). Data are expressed as the mean ± SD. Statistical significance was determined by a two‐tailed unpaired *t*‐test for total RNA, *p* = significant differences from control. Correlations were calculated using Pearson's product–moment correlation coefficients.

The Yoshida AH‐130 tumor is an established model of hepatoma‐associated cancer cachexia (Tessitore et al., 1987). Inoculated rats developed tumors with a mean volume of 62.68 ± 3.92 mL, and an average cell count of 5877.00 ± 279.13, and resulted in a significantly lower final tumor‐free body weight (FBW) relative to initial body weight (IBW) when compared to controls (*p* < 0.001; Figure 1a). Tumor inoculated rats experienced a 25.22% loss of skeletal muscle (*p* < 0.001; Figure 1b) and a 17.75% loss of heart mass (*p* < 0.001) relative to IBW (Figure 1c). In skeletal muscle, rRNA was 39.54% (*p* = 0.002) lower in tumor‐bearing rats (Figure 2a), and 36.48% (*p* = 0.074) lower in the heart when compared to controls (Figure 2b). The reduction in rRNA was significantly correlated with muscle weight (*r* = 0.628, *p* = 0.021; Figure 2a) and cardiac weight (*r* = 0.557, *p* = 0.048; Figure 2b).

### 3.2 Yoshida AH‐130 ascites hepatoma modulate translational initiation factor phosphorylation in both skeletal and cardiac muscle

**FIGURE 3:**
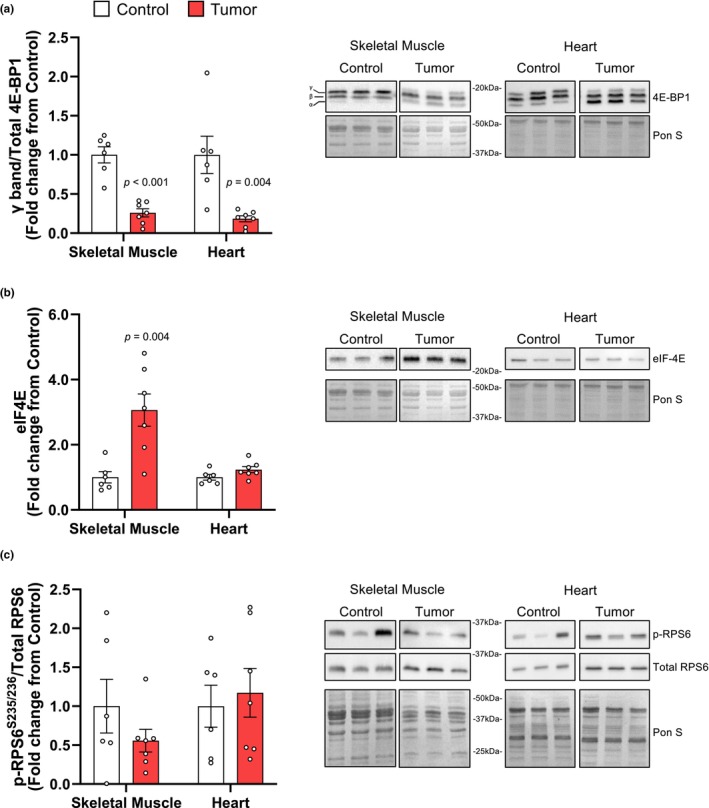
*4E‐BP1 is hypophosphorylated in both the skeletal and heart muscle of tumor‐bearing rats. A:* 4E‐BP1 quantification, the 4E‐BP1γ isoform was normalized to total 4E‐BP1 to assess changes in protein phosphorylation. B) Quantification of eIF4E, total eIF4E was normalized to Ponceau S. C) Phosphorylated ribosomal protein S6 at S235/236 (p‐RPS6S235/236) was normalized to total RPS6. Representative images of all Western blots (10mg of protein) are accompanied by the corresponding Ponceau S (Pon S) staining. Data are expressed as mean fold‐change from controls ± SD. Statistical significance was determined by a two‐tailed unpaired *t*‐test, *P* = significant differences from control.

In addition to a reduction in translational capacity, the Yoshida AH‐130 ascites hepatoma caused a significant reduction in 4E‐BP1 phosphorylation in both skeletal (−74.02%, *p* < 0.001) and cardiac (−81.66%, *p* = 0.004) muscles (Figure 3a,b). The eIF4E protein levels were elevated in the skeletal muscle of AH‐130 tumor rats (206.20%, *p* = 0.004; Figure 3a), but no difference was found in the heart (Figure 3b). No significant differences in RPS6 phosphorylation (Ser 235/236) were found in either skeletal muscle or heart (Figure 3a,b).

### 3.3 UPR gene expression is elevated in Yoshida AH‐130 treated rats

**FIGURE 4:**
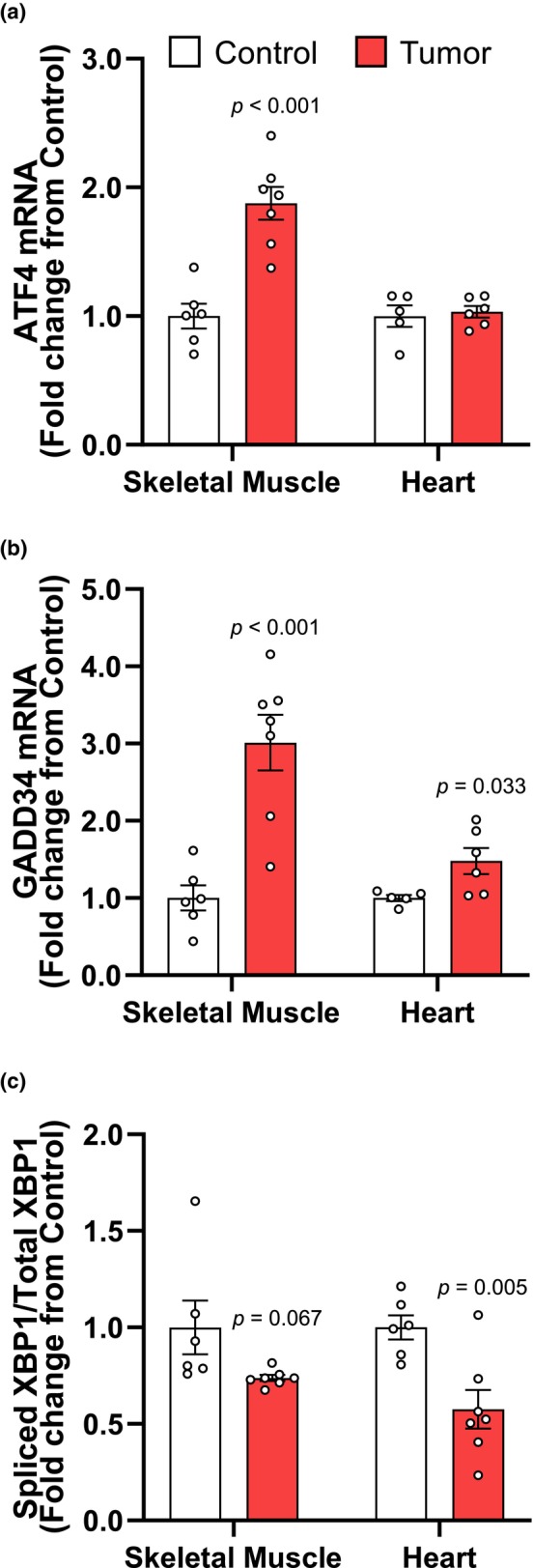
Expression of ER‐stress mRNA suggests dysregulation of the UPR in the skeletal and heart muscles of Yoshida AH‐130 rats. A: expression of ATF4, B) GADD34, and C) spliced XBP‐1 normalized to total XBP‐1 mRNA in skeletal muscle and heart. Data are presented as mean fold‐change from controls ± SEM. Statistical significance was determined by a two‐tailed unpaired *t*‐test, *P* = significant differences from control.

Tumor presence increased mRNA levels of ATF4 (87.76%, *p* < 0.001) and GADD34 (301.20%, *p* < 0.001) in skeletal muscle (Figure 4a) but only GADD34 mRNA expression was induced in the heart (48.10%, *p* = 0.033; Figure 4b). XBP‐1 splicing (s‐XBP1) was significantly lower in both the skeletal (−26.14%, *p* = 0.067) and cardiac (−42.44%, *p* = 0.005) muscles of tumor‐bearing rats (Figure 4a,b).

### 3.4 Yoshida AH‐130 induced skeletal and cardiac muscle wasting involve the expression of local pro‐inflammatory cytokines

**FIGURE 5:**
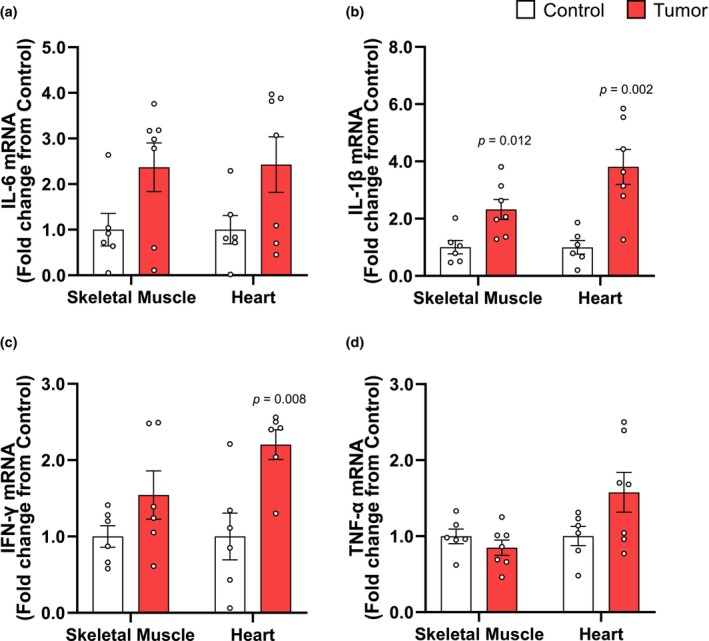
Local tissue expression of inflammatory mRNA is increased in cachectic rats. (a) Expression of IL‐6, (b) IL‐1β, (c) interferon‐gamma (IFN‐γ), and (d) TNF‐α mRNA in skeletal muscle and heart. Data are presented as mean fold‐change from controls ± SEM. Statistical significance was determined by a two‐tailed unpaired *t*‐test, *P* = significant differences from control.

Following tumor inoculation, tissue IL‐6 mRNA levels were on average higher in skeletal (136.70%, *p* = 0.065) and cardiac (142.60%, *p* = 0.073) muscle, respectively (Figure 5a,b). In the skeletal muscle, IL‐1β mRNA expression was elevated in the tumor group by 79.55% (*p* = 0.012; Figure 5a), and by 116.90% (*p* = 0.002) in the heart (Figure 5b). Average IFN‐γ mRNA in the skeletal muscle was not different from control, but it was significantly elevated in the heart by 75.12% (*p* = 0.008; Figure 5b). TNF‐α mRNA levels in skeletal muscle were not affected by the tumor, but heart levels increase on average without being statistically significant (Figure 5a,b).

## 4 DISCUSSION

We investigated changes in skeletal and cardiac muscle mass in the Yoshida AH‐130 rat hepatoma tumor model. Inoculation with tumor cells resulted in lower skeletal and cardiac muscle mass, anabolic deficits, increased UPR and tissue expression of pro‐inflammatory cytokines. Prior studies (Baracos et al., 1995; Poetsch et al., 2023; Springer et al., 2014; Toledo et al., 2014) using this model showed that skeletal muscle loss is associated reductions in muscle strength and daily activity within 1 week of tumor implantation (Poetsch et al., 2023; Toledo et al., 2011). Similarly, a reduction in left ventricular mass is consistent with lower ejection fraction, stroke volume, heart rate, and decreased survivability (Poetsch et al., 2023; Springer et al., 2014; Toledo et al., 2014). Thus, the reduction in skeletal and cardiac mass in the present study is consistent with previously reported functional deficits. In agreement with our previous studies in ovarian, lung, and colon cancer models (Belcher et al., 2022; Kim et al., 2021, 2022), the Yoshida AH‐130 tumors caused a reduction in translational capacity in both skeletal and cardiac muscle, although the decline in the heart did not reach statistical significance at the time point studied. These findings align with earlier investigations using this model (Baracos et al., 1995), and support the interpretation that anabolic deficits may play a central role in skeletal and cardiac muscle wasting (Belcher et al., 2022; Bhogal et al., 2006; Kim et al., 2021, 2022). While the loss of skeletal muscle is well defined in cachexia, the impact of this condition on cardiac anabolism is less clear. In this model, cardiac rRNA levels were reduced by ~36% following tumor inoculation. Despite not achieving statistical significance, the reduction in translation capacity strongly correlated with the alterations in cardiac mass, indicating that like in skeletal muscle, the loss of ribosomes may play a critical role in cardiac muscle wasting. Another finding implicating defective protein anabolism is the modulation of cap‐dependent translation factors. Similar to other models of cancer cachexia (Brown et al., 2018; Manne et al., 2013; White et al., 2011), Yoshida tumors caused a significant decrease in 4E‐BP1 phosphorylation in both skeletal and cardiac muscle. 4E‐BP1 hypophosphorylation increases its binding affinity for eIF4E, prevents the formation of the translation initiation complex eIF4F and reduces protein synthesis (Gingras et al., 1999). In contrast with 4E‐BP1 hypophosphorylation, skeletal muscle eIF4E protein levels were significantly elevated in tumor‐bearing rats, a response that was not mirrored in the heart. This may represent a feedback mechanism attempting to restore proteostatic balance that is operant in skeletal but not in cardiac muscle. Together, these findings indicate that Yoshida AH‐130 tumors negatively affect the protein synthesis machinery and suggest that both translational capacity and efficiency may underlie the anabolic deficits characteristic of tumor‐induced wasting.

The alterations in translational control mechanisms lead us to investigate the UPR. As expected, we observed an increase in ATF4 and GADD34 mRNA levels in skeletal muscle, however only GADD34 was elevated in the heart of tumor‐bearing rats. These findings partially align with prior observations showing an increase in ATF4 mRNA levels in skeletal muscle of LLC mice (Bohnert et al., 2019), while GADD34 expression remained unchanged (Bohnert et al., 2016). This raises the question as to what extent the UPR is conserved across cachexia models or whether different tumors provoke a specific UPR. We also examined changes in XBP1 splicing and found a robust reduction in sXBP1 in both skeletal and cardiac muscle, although it did not achieve significance in skeletal muscle. Contrary to the findings in this model, an increase sXBP1 has been previously reported in skeletal muscle of cachectic LLC mice (Bohnert et al., 2016, 2019). This is intriguing because XBP1 is spliced under cellular stress (i.e. with activation of the UPR) (Bohnert et al., 2019). We interpret this discrepancy as the disruption of the UPR being a possible atrophy exacerbating factor. A failure to mount the UPR has been shown to increase muscle wasting, LLC mice treated with the UPR inhibitor 4‐Phenylbutyric acid (4‐PBA) exhibited a suppressed ER stress response and aggravated muscle wasting. Similarly, myotubes incubated with LLC conditioned media +4‐PBA displayed a smaller diameter and increased activation of proteolytic markers (Bohnert et al., 2016). Thus, a defective UPR activation may result in failure to restore proteostasis in this hepatoma tumor model.

Skeletal and cardiac muscle atrophy in cancer has often been linked to changes in systemic pro‐inflammatory cytokines (Bordignon et al., 2022; Rausch et al., 2021; Webster et al., 2020), which can stimulate the local expression of pro‐inflammatory effectors of muscle wasting (i.e. cytokine‐induced cytokine‐release) (Catalano et al., 2003; Luo et al., 2003; Podbregar et al., 2013). We found an increase in IL‐6 mRNA in both skeletal muscle and heart of cachectic rats, but statistical significance was not achieved likely due to experimental variability associated with the Yoshida hepatoma model. The increase in IL‐1β mRNA was similar in skeletal and cardiac muscle. Within skeletal muscle, IL‐1β can exert direct autocrine effects and activate the NF‐κB pathway, leading to increased protein breakdown and muscle wasting (Cai et al., 2004). The increase in IL‐1β was also evident in the heart indicating a possible conserved response of striated muscle to the tumor. Increased expression of IL‐1β in cardiomyocytes of rats with chronic heart failure leads to substantial interstitial fibrosis and impaired cardiac function (Shioi et al., 1997). Thus, the increase in IL‐1β expression in response to tumors may reflect a common response to systemic inflammation. We also observed a significant increase in IFN‐γ mRNA expression in the cardiac muscle where it can cause impairments in contractility by acting in an autocrine fashion (Castro et al., 2018; Curtsinger et al., 2012). Changes in TNF‐α were evident and variable but not significant in the skeletal muscle, and are contrary to a previous report indicating elevated TNF‐α mRNA in skeletal muscle of rats bearing Yoshida hepatoma tumors (Catalano et al., 2003). Overall, these findings suggest that skeletal and cardiac muscle display divergent patterns of pro‐inflammatory cytokine expression despite undergoing similar atrophy, and that these factors may mediate tissue‐specific cancer‐related alterations in physiological functions.

## 5 SIGNIFICANCE AND LIMITATIONS

In summary, we provide evidence demonstrating that impaired anabolism, UPR, and tissue production of pro‐inflammatory cytokines may converge to facilitate tumor‐induced reductions in skeletal and cardiac muscle mass. Consistent alterations in translational capacity and efficiency, with divergent proteostatic stress control, and tissue pro‐inflammatory cytokine expression in the Yoshida model of hepatocellular carcinoma highlight the need for a better understanding of the molecular mechanisms involved in cancer cachexia. Our interpretations of the results need to be contextualized within a few limitations of the study: for example, the age of the rats, which represent early adulthood at (8–9 weeks of age) (Sengupta, 2013), may involve slightly different mechanisms than those in older animals. The short course of the model (7 days) is substantially shorter than the human disease course, 11.8 days in early rat adulthood corresponds to one human year (Sengupta, 2013). Thus, the 7‐day period in our study translates to approximately 3 months in humans. Because the median survival following diagnosis of hepatocellular carcinoma in humans is ~6–20 months (Golabi et al., 2017), the present model is substantially more aggressive than the reported human pathology. Survival rates are typically lower in male hepatocellular carcinoma patients (Rich et al., 2020) and males are more susceptible to develop cachexia (Rich et al., 2022), so understanding how the reported mechanisms are conserved in female rats warrants further research.

## AUTHOR CONTRIBUTIONS

S.B. and G.A.N. conceived and designed the study, D.J.B., N.K., B.N.‐L., M.M., F.J.L.‐S. performed the experiments; D.J.B. and N.K. analyzed the data; D.J.B., N.K., and G.A.N. interpreted results; D.J.B. and N.K. drafted the manuscript; D.J.B., N.K., F.J.L.‐S., S.B., and G.A.N. revised manuscript. All authors approved the final version of the manuscript.

## FUNDING INFORMATION

This study was supported by grant AR078430 from the National Institutes of Health (NIH) to G.A.N., and grant SAF2015‐65589‐P from the Ministerio de Economia y Competitividad to S.B.R.

## CONFLICT OF INTEREST STATEMENT

The authors declare no conflicts of interest, financial or otherwise.

## Data Availability

The data that support the findings of this study are available from the corresponding author upon reasonable request.
